# Washed Microbiota Transplantation Accelerates the Recovery of Abnormal Changes by Light-Induced Stress in Tree Shrews

**DOI:** 10.3389/fcimb.2021.685019

**Published:** 2021-06-23

**Authors:** Jing Wang, Qianqian Li, Qi Huang, Meng Lv, Pan Li, Jing Dai, Minjie Zhou, Jialu Xu, Faming Zhang, Jun Gao

**Affiliations:** ^1^ Department of Neurobiology, School of Basic Medical Sciences, Nanjing Medical University, Nanjing, China; ^2^ Visual Cognition Laboratory, Department of Medicine, University of Fribourg, Fribourg, Switzerland; ^3^ Medical Center for Digestive Diseases, The Second Affiliated Hospital of Nanjing Medical University, Nanjing, China; ^4^ Key Lab of Holistic Integrative Enterology, Nanjing Medical University, Nanjing, China; ^5^ PET Center, Huashan Hospital, Fudan University, Shanghai, China; ^6^ Animal Core Facility of Nanjing Medical University, Nanjing Medical University, Nanjing, China; ^7^ Department of Rehabilitation Medicine, Jiangsu Shengze Hospital Affiliated to Nanjing Medical University, Nanjing Medical University, Nanjing, China

**Keywords:** gut–brain axis, staying up late, stress, tree shrew, magnetic resonance imaging, washed microbiota transplantation

## Abstract

The gut and brain interact constantly in a complex fashion. Its intricacy and intrigue is progressively being revealed in the study of the “gut–brain axis”. Among many factors, abnormal light exposure is a potential powerful stressor, which is becoming ever more pervasive in our modern society. However, little is known about how stress, induced by staying up late by light, affects the gut–brain axis. We addressed this question by extending the normal circadian light for four hours at night in fifteen male tree shrews to simulate the pattern of staying up late in humans. The behavior, biochemical tests, microbiota dynamics, and brain structure of tree shrews were evaluated. The simple prolongation of light in the environment resulted in substantial changes of body weight loss, behavioral differences, total sleep time reduction, and an increased level of urine cortisol. These alterations were rescued by the treatment of either ketamine or washed microbiota transplantation (WMT). Importantly, the sustainability of WMT effect was better than that of ketamine. Magnetic Resonance Imaging analysis indicated that ketamine acted on the hippocampus and thalamus, and WMT mainly affected the piriform cortex and lateral geniculate nucleus. In conclusion, long-term light stimulation could change the behaviors, composition of gut microbiota and brain structure in tree shrews. Targeting microbiota thus certainly holds promise as a treatment for neuropsychiatric disorders, including but not limited to stress-related diseases.

## Introduction

The intricate interplay of gut and brain is quickly and surely revealing its implications to many facets of human health, including stress-related neural diseases (Gilbert et al., 2018; Cryan et al., 2019; Zhang et al., 2019; Pennisi, 2020). Any disturbance of this system could so lead to ill-fated effects. Like the spreading pervasive presence of artificial light. It has, on one side, drastically increased humans’ productivity on a societal scale and distinctly enriched parts of their Umwelt in a stimulating manner from La Ville Lumière to Times Square. Otherwise, it’s also a potential powerful stressor and disturbing factor for organisms and their well-being. Recent studies have demonstrated that exposure to light at night had negative influences on gut microbiota. Benedict et al. observed that two days of recurrent partial sleep deprivation, that is, only staying up late for 4 h daily, caused changes in gut microbiota of young adults (Benedict et al., 2016). Additionally, excessive light exposure affects mood and brain circuits, such as the suprachiasmatic nucleus of the hypothalamus, medial amygdala, lateral habenula and hippocampus (Bedrosian and Nelson, 2017). However, it is currently unclear how long-term staying up late affects the gut–brain axis. To answer this question, we need a suitable animal model to better mimic our modern predicament.

The omnivorous tree shrew (*Tupaia belangeri*) is a highly qualified candidate model organism for such aims. First and foremost the tree shrew has a close genetic relationship with primates and resembles the pattern of human sleep on a more fundamental level than rodents do, due to being day-active and having longer, more continuous bouts instead of the fractured, polyphasic, nocturnal nature of rodent sleep (Liu et al., 2001; Coolen et al., 2012; Fan et al., 2013). Second, tree shrews have a small size (150–170 g), a short gestation (43 days) and a rapid sexual maturity (3–6 months), which facilitates their ease of use as a laboratory species next to rats and mice. Third, tree shrews have established their place in the study of a wide variety of neurological diseases, including psychosocial stresses, visual cognition and depression as a new primate-like animal organism (Wang et al., 2013a; Schmelting et al., 2014; Khani et al., 2018). Meanwhile, tree shrew is highly vulnerable to stress which makes them a promising stress model (Fang et al., 2016). Therefore, we developed here a staying up late model in tree shrews. It was evaluated by measuring physiological and biochemical indicators, combined with characterization of gut microbiota and brain structure through magnetic resonance imaging (MRI).

To ascertain whether targeting gut microbiota could reverse the changes in brain structure and ill-health Washed Microbiota Transplantation (WMT) was used. Given that gut microbiota can have a major impact on brain function and behavior through the gut–brain axis (Vendrik et al., 2020). WMT is a modification of Fecal Microbiota Transplantation (FMT), where the manual manipulations are substituted by a more automated microbiota purification system and washing process. FMT has extremely high efficacy in recurrent or refractory *Clostridium difficile* infection with the ability to restore healthy microbial ecology, and thus holds promise as a therapy for other diseases influenced by dysbiosis of the intestinal environment (Zhang et al., 2020). Additionally, WMT is demonstrated to be safer, more precise and more quality-controllable than the crude FMT (Zhang et al., 2020). Converging evidence suggests that both germ-free and antibiotic treatment have an impact on animal stress (Sudo et al., 2004; Gareau et al., 2011; Peirce and Alvina, 2019; Wang et al., 2020). This could make results from germ-free animals more difficult to interpret and translate. Considering that this is a stress animal model, healthy tree shrews were chosen without any antibiotic treatment. Our study aimed to evaluate the alterations of behavioral and brain structures after gut microbiota reconstruction using WMT. We hypothesize that WMT could ameliorate the physiological changes and accelerate the recovery of abnormal structural changes in the brain induced by extended light exposure to tree shrews.

## Materials and Methods

### Animals

Adult male and female Chinese tree shrews (*T. belangeri Chinensis*, N = 18) weighing 110–160 g were obtained from the Kunming Institute of Zoology, Chinese Academy of Sciences. We used fifteen male tree shrews for all behavioral experiments and three females, who were isolated from males to avoid ovulation, as the negative controls in MRI. All animals were bred at the Animal Core Facility of Nanjing Medical University. They were given *ad libitum* access to food and water. Each tree shrew was housed individually in climate-controlled rooms (ambient temperature: 25–27°C, air humidity: 55–70%) under a 12 h light/12 h dark cycle (light, 08:00–20:00; dark, 20:00–08:00) in the baseline phase. All animal experiments were performed in accordance with the recommendations of the Experimental Animal Ethics Committee at the Nanjing Medical University. The related fecal donors’ samples were approved by institutional committees.

### Experimental Procedures

**Figure 1:**
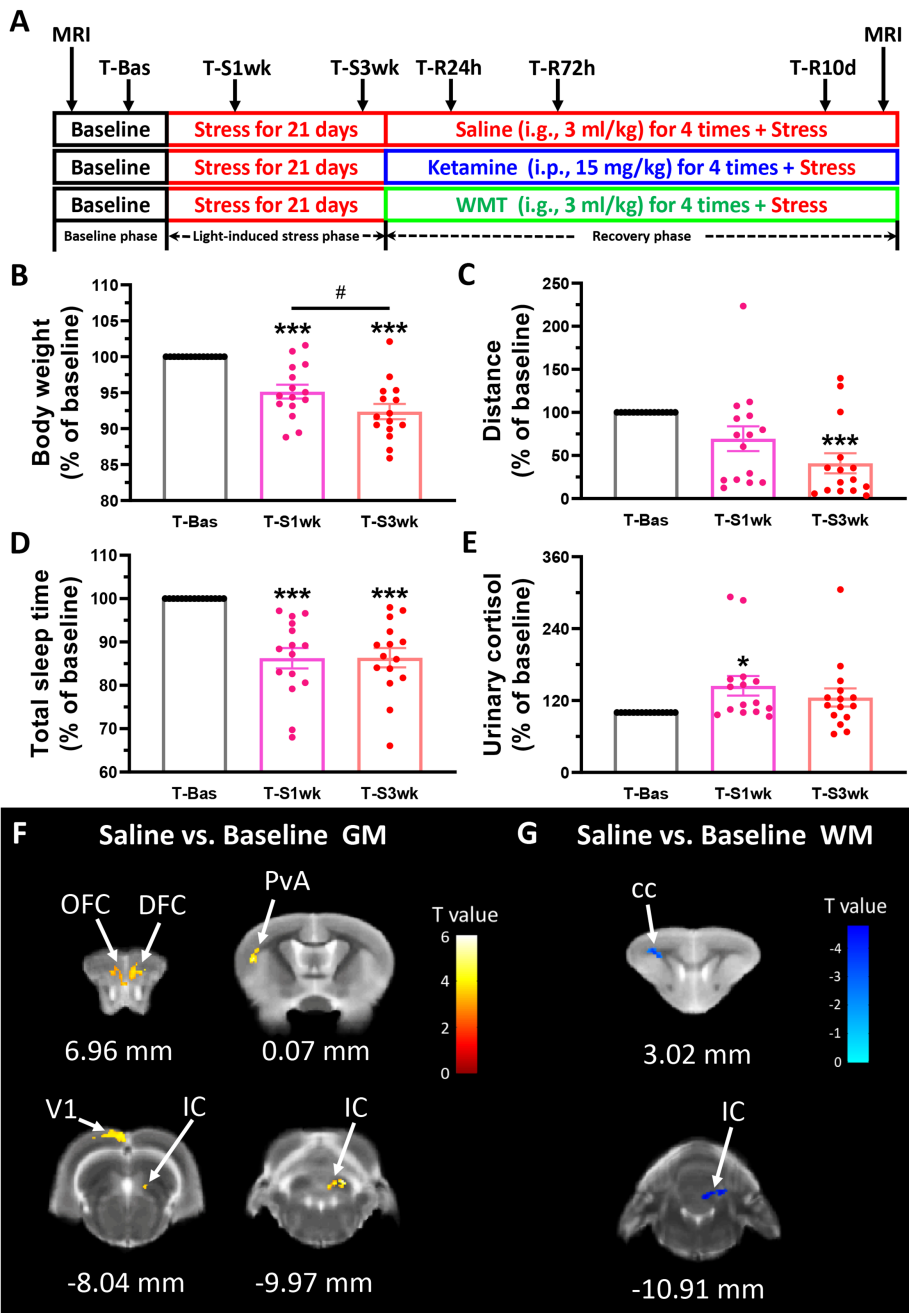
Physiological responses and brain structure changes in the tree shrew after staying up late for 21 days. **(A)** Experimental design. Basic data was collected in the baseline phase (T-Bas), including MRI scanning, weight, urinary and stool collection, locomotor activity and total sleep time measurements. In the light-induced stress phase, the facility light cycle was changed (light on at 8:00, light off at 24:00). All of the above indicators, except MRI scanning, were tested extended light exposure for one week (T-S1wk) and three weeks (T-S3wk). Ketamine (15 mg/kg), WMT (3 ml/kg) or vehicle (0.9% saline, 3 ml/kg) were administrated at the beginning of the recovery phase for a total of four times. We tested all physiological and biochemical indicators in the ketamine, WMT or vehicle (saline) conditions 24 h (T-R24h), 72 h (T-R72h) and 10 days (T-R10d) after administration. At the end of the experiment, MRI scans were performed on the same animals. **(B**–**E)** All of the physiological and biochemical indicators after light-induced stress for 1 week (T-S1wk) and 3 weeks (T-S3wk) were normalized with their values in the baseline phase (T-Bas) to explore the effects of extended and prolonged light exposure. Body weight (**B**, N = 15), distance travelled in an open field box (**C**, N = 15) and total sleep time (**D**, N = 15) were all decreased, and morning urinary cortisol (**E**, N = 15, [F(42,2) = 3.025, p = 0.059], LSD Post Hoc: T-S1wk compared with T-Bas, p = 0.018) was increased after staying up late for 21 days. **(F)** The GM density signals from OFC (orbital frontal cortex), DFC (dorsal frontal cortex), PvA (parietal ventral area), V1 (primary visual cortex) and IC (inferior colliculus) were all increased (N = 3/group). **(G)** The WM density signals from cc (corpus callosum) and IC were decreased (N = 3/group). Error bars show the SEM. *means compared with control in baseline phase, *p <0.05, ***p <0.001. # means compared between each other in stress phase, #p <0.05.

As shown in **Figure 1A**, the whole experiment included; a baseline phase (7 days), a light-induced stress phase (21 days) and a recovery phase (10 days). In the first phase, the baseline phase (T-Bas) we measured a range of physiological and biochemical indicators, including body weight, locomotion, morning urine cortisol, and total sleep time. Fecal samples of the tree shrews were collected for 16S ribosomal RNA (rRNA) screening. Nine male and three female animals were randomized and scanned with MRI to study brain structure changes. Then in the second phase, we prolonged environmental light until later in the evening by 4 h, that is, the 12 h light/12 h dark cycle was changed to a 16 h light/8 h dark cycle (light, 08:00–24:00; dark, 00:00–08:00). We did not force the animals to keep awake till 24:00 but simply left the light on. During this light-induced stress phase, all physiological and biochemical indicators were measured in the first and third weeks (T-S1wk and T-S3wk). In the last recovery phase, we maintained the 16 h light/8 h dark cycle for the tree shrews and divided all animals into three groups of five: Saline, Ketamine and WMT. The microbiota for WMT was from one healthy donor in China Microbiota Transplantation System for the treatment condition. The methodology WMT based on an automatic microbiota purification system (GenFMTer, Nanjing, China) followed by centrifugation plus suspension for three times in a specifically designed laboratory at good manufacture practice (GMP) level (Zhang et al., 2020). All tree shrews were treated with WMT (~1.0 × 101^3^ bacteria/ml colony-forming units, intragastric administration, i.g., 3 ml/kg) or ketamine (intraperitoneal injection, i.p., 15 mg/kg) or vehicle (0.9% sterile saline, i.g., 3 ml/kg). To reinforce the donor microbiota efficacy, drug or vehicle was administered four times: at 08:00 and 20:00 of the first day and 20:00 of the second and third days. We measured all physiological and biochemical indicators at 24 h, 72 h and 10 days (T-R24h, T-R72h and T-R10d) after the first administration. Finally, the brain structures of the animals scanned in the baseline phase were scanned again with MRI at the end of our experimental timeline. Following the experiments, tree shrews were put back, and sedated with diethyl ether to then be euthanized by rapid decapitation. The brain was quickly removed to be prepared and stored for future experiments.

### Measurements

#### Body Weight and Analysis of Urinary Cortisol

Tree shrews were weighted between 7:45 and 08:00 before breakfast. Simultaneously urine samples were collected. Urine samples were stored at −20°C until analysis and free cortisol was measured by Access Immunoassay System (Unicel DxI 800, Beckman Coulter, Inc., USA).

#### 16S rRNA Gene Sequencing and Processing

Fresh fecal samples of tree shrews were collected between 8:45 and 09:15 after breakfast. All of the fecal samples that were used for WMT, originated from healthy humans, who donated to the Chinese fecal microbiota bank (fmtBank). All donors provided written informed consents prior to participation in this study. This study was reviewed and approved by the Second Affiliated Hospital of Nanjing Medical University Institutional Review Board. Samples were stored at −80°C before the eventual analysis. Microbial DNA was extracted from stool samples. Bacterial 16S rRNA gene sequences were PCR amplified using bar-coded primers for the V4–V5 hypervariable region by the Phusion High-Fidelity PCR Master Mix with HF buffer (New England Biolabs, England). Products from each sample were mixed at equal molar ratios and then sequenced using the Illumina MiSeq platform (Illumina, Inc., San Diego, CA, USA), following standard Illumina sequencing protocols. Operational taxonomic units (OTUs) were clustered at 97% similarity and filtered using the UPARSE pipeline. Unweighted UniFrac distances were visualized with principal coordinate analysis (PCoA) using Python.

#### Locomotor Behavior Analysis

The open field box (length × width × height = 50 cm × 50 cm × 80 cm) was made of polymethyl methacrylate sheets (plexiglass), with one transparent side that faces the camera and the other three white and opaque. After all samples are collected, tree shrews were put into the open field box for 15 min. An Any-maze Animal Behavior Video Analysis System (ST-60000, Sterling, USA) was set to analyze the distance travelled in the box for evaluation of locomotor behavior.

#### Total Sleep Time

To monitor the sleep of the tree shrews, an infrared night-vision pinhole camera in the nest box was installed before the whole procedure. The monitoring started at 20:00 and ended at 08:30 the next morning. The time of the animals fell asleep and woke up was manually calculated by persons not involved in the experimental design nor drug administration for avoiding bias.

### Magnetic Resonance Imaging

#### *In Vivo* MRI Scanning

All *in vivo* MRI scans were carried out on a 7.0T animal MRI scanner (Biospec 7T/20 USR, Bruker BioSpin GmbH, Germany). Nine of fifteen animals involved in locomotor analysis and three naïve animals were selected for MRI. Tree shrews were fasted overnight before each scanning session. Before placing them in a prone position on the scanning bed, we anesthetized them with isoflurane (5% for induction and 1.5–2.0% for maintenance) (Huang et al., 2018). T2-weighted anatomical images were acquired with Rapid Acquisition with Relaxation Enhancement (RARE) sequence (RARE factor = 8, repetition time = 2838.2 ms, echo time = 33 ms, matrix size 256 × 256 × 27, voxel size 0.06 × 0.04 × 1.00 mm^3^, no slice gap). All the Bruker original images were converted to the DICOM format with software programs (Paravision 4.0) in the scanner.

#### MRI Data Processing

Voxel-based morphometry analysis was performed using MATLAB R2014a and SPM12 (Ashburner and Friston, 2000). First, the voxel size of all images was scaled by a factor of six to better approximate the human size. Then the images were spatially normalized into the stereotaxic space (Ni et al., 2016; Huang et al., 2018) and segmented into grey matter (GM) and white matter (WM) probability maps using the unified segmentation approach (Ashburner and Friston, 2005). After that, the maps were smoothed by a Gaussian kernel of three times of its voxel size. Finally, a two-sample t test was conducted to compare the difference between saline/ketamine/WMT and baseline group, as well as ketamine and WMT group, for GM and WM density respectively. In the statistical analysis, voxels with a value of <0.2 were excluded to avoid possible edge effects around the borders between the tissue classes and to include only voxels with sufficient tissue class proportions. The statistical significance was set at p <0.01 and a cluster extent threshold of 155 voxels, where no significant difference survived between the negative controls.

### Statistical Analysis

All data were analysed using SPSS 19.0 (SPSS, Inc., Chicago, IL, USA) or GraphPad (version 5; GraphPad Software, San Diego, CA, USA). Wilcoxon rank-sum test, independent t test and paired-samples t test were used to analyze differences between two groups. More than two groups were analyzed by one-way ANOVA test followed by LSD Post Hoc test. A probability level less than 0.05 was considered as statistical significance except for MRI analysis.

## Results

### Physiology, Behaviors and Brain Structure of Tree Shrews Were Altered After Staying Up Late by Light Exposure

An overview of the experimental procedure timeline can be seen in **Figure 1A**. Increased light exposure until midnight for 21 days alters physiological parameters of tree shrews. One-way ANOVA analysis showed a remarkable decrease in body weight [F(42,2) = 21.572, p <0.001] and distance travelled in 15 min [F(42,2) = 7.638, p = 0.001] after three weeks of extended light exposure (**Figures 1B, C** **)**. A LSD Post Hoc analysis revealed that body weight was more negatively affected by light-induced stress than locomotor behavior. It has already a significant decrease after 1 week (p <0.001), and this decrease continued downwards into the third week (p <0.001 compared to baseline, p <0.05 compared to T-S1wk). The timing of sleep onset and waking up was monitored by an infrared night-vision pinhole camera in their next box. During the baseline phase, the animal usually woke up around 7:44 before the light turned on and fell asleep around 20:06 after the light turned off (**Table S1**). However, after extended light exposure, the total sleep duration of the tree shrews decreased significantly as seen in infrared monitoring [F(42,2) = 17.910, p <0.001, **Figure 1D**]. This was mostly due to a delayed sleep onset (**Table S1**). Cortisol, a major stress hormone released by the adrenal gland, has been implicated in stress-related diseases when levels are chronically elevated (Pariante and Lightman, 2008). In tree shrews, like in humans, cortisol but not corticosterone is the main stress-related hormone (Wang et al., 2013b). Compared to their baseline, they exhibited a higher level of morning urinary cortisol in the first week (p <0.05, **Figure 1E**).

For each group we performed two MRI scans: one at the beginning of the experiment when the tree shrews are still housed in a normally lighted environment (T-Bas), and another after the recovery phase (the day after T-R10d). The alterations in brain structure of the tree shrews in the saline group were considered to be caused solely by light-induced stress. Comparing between the saline group and the baseline, results demonstrated that there was a significant increase in the density of gray matter (GM) in the frontal cortex, somatosensory cortex, visual cortex and thalamus after staying up late, and a robust decrease in white matter (WM) density in the corpus callosum and thalamus (**Figures 1F, G** and **Table 1**). The behavioral and cortisol tests indicated that stress was successfully induced across groups, and that tree shrews that stay up longer at night due to light have alterations in the structure of their brain.

**Table 1:** Brain regions of GM and WM density change after staying up late in saline group compared with baseline phase.

Brain region	Cluster location	Coordinate			Cluster size (mm^3^)	T value	P
		x	y	z			
**Increase**							
Frontal cortex	**DFC**	−3	4	7	7.97	4.67	0.000^***^
Somatosensory cortex	**PvA**	6	5	0	8.91	5.06	0.000^***^
Visual cortex	**V1**	1	1	−8	2.64	4.16	0.001^**^
Thalamus	**IC**	−2	7	−10	2.67	5.96	0.000^***^
**Decrease**							
Corpus callosum	**cc**	4	3	3	3.47	3.99	0.001^**^
Thalamus	**IC**	−2	7	−11	3.23	4.64	0.000^***^

Cluster location represented for the subregion of maximum t-value in the brain region according to the fine stereotaxic brain atlas of the tree shrew (Zhou and Ni, 2016). Coordinates (x, y, z): the coordinates in 3D stereotaxic coordinate system accordant with the histological atlas. Cluster size: the volume of a cluster. T value: the maximum t-value in each cluster. P, the maximum confidence level in each cluster. **p <0.01, ***p <0.001.

### Gut Microbiota of Tree Shrews Were Changed After Extended Light Exposure

**Figure 2:**
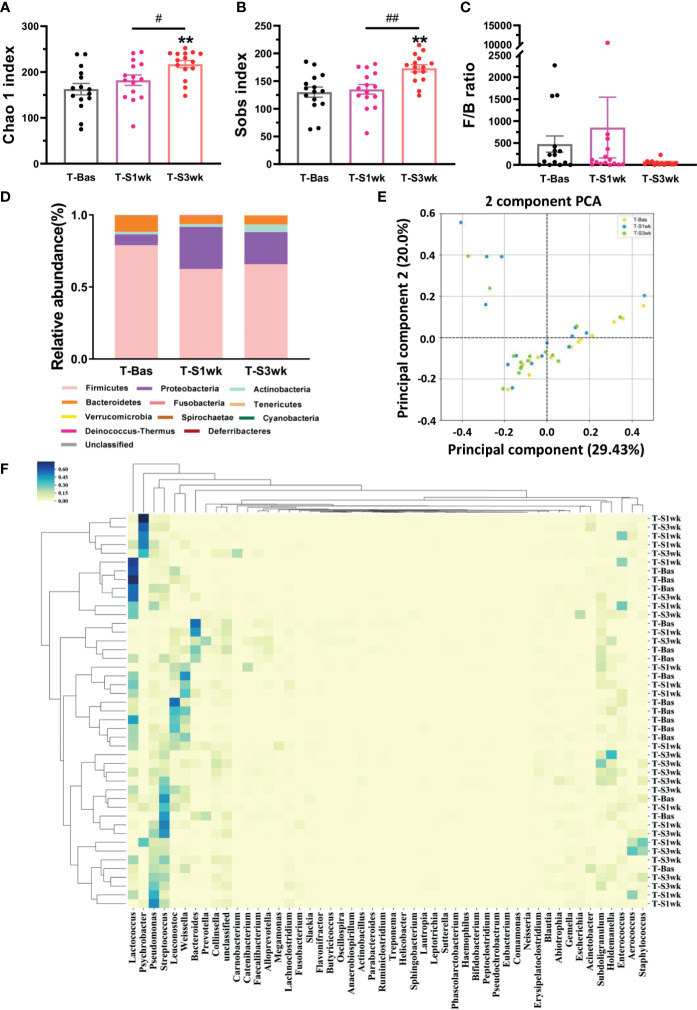
The variations of gut microbiota composition and biodiversity after staying up late for 21 days in tree shrews. **(A)** The Chao 1 index significantly increased at T-S3wk in light-induced stress phase (LSD Post Hoc: compared with T-Bas, p = 0.001, compared with T-S1wk, p = 0.026). **(B)** The Sobs index showed an increased level at T-S3wk in light-induced stress phase (LSD Post Hoc: compared with T-Bas, p = 0.001, compared with T-S1wk, p = 0.002). **(C)** The ratio of *Firmicutes*-to-*Bacteroidetes* (F/B ratio) increased at T-S1wk but decreased at T-S3wk. **(D)** The median relative abundance of the *Firmicutes* and *Bacteroidetes* decreased but that of the *Proteobacteria* and *Actinobacteria* increased. **(E)** The PCA on gut microbiota community of the tree shrews at baseline was different from that in the third week after stress. Each dot stands for one sample. **(F)** The heat map presented the microbial distribution of the tree shrews at baseline was different from that in the first week and the third week after stress. N = 15. Error bars show the SEM. **means compared with control in baseline phase, **p <0.01. # means compared between each other in stress phase, #p <0.05, ##p <0.01.

Previous studies indicated that stress could perturb the composition of the gut microbiota and affect host behavior (Foster et al., 2017). Thus, we collected the fecal samples and performed gut microbiota 16S rRNA gene sequencing in tree shrews before and after introducing extended environmental lighting. As shown in **Figure 2**, the α-diversity of the gut microbiota increased significantly after 21 days of extended light exposure [Chao 1 index: F(42,2) = 6.619, p = 0.003; Sobs index: F(42,2) = 8.245, p = 0.001]. Especially in the third week, both of them had a significant increase compared with the baseline (T-Bas) or the first week test (T-S1wk) (**Figures 2A, B** **)**. We observed a trend where the *Firmicutes*-to-*Bacteroidetes* ratio went up and down (**Figure 2C**). The median relative abundances of the *Proteobacteria* and *Actinobacteria* increased along a decrease in *Firmicutes* and *Bacteroidetes* (**Figure 2D**). The β-diversity of the gut microbiota, described by principal components analysis (PCA) and represented in the heat map, revealed a remarkable alteration after extended environmental illumination (**Figures 2E, F** **)**. Further correlation analysis between changes of microbiota and alterations of physiological responses (**Table S2**) demonstrated that targeting microbiota could be a potential method of treatment for neuropsychiatric disorders.

### Basic Gut Microbiota Structure Showed Similarities and Differences Between Healthy Humans and Tree Shrews

**Figure 3:**
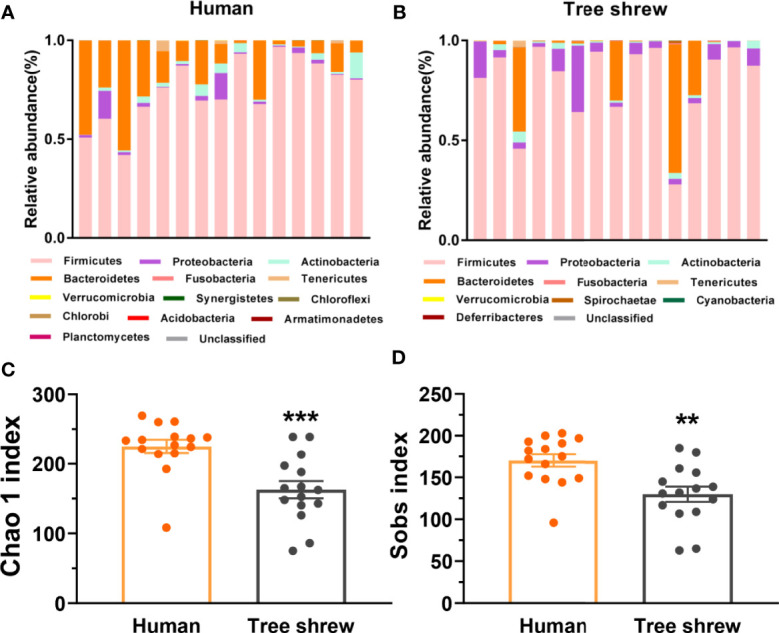
Differential profiles of the gut microbiota composition and α-diversity between healthy humans and tree shrews. **(A, B)** The bar charts of gut microbiota abundance of healthy humans (**A**, N = 15) and tree shrews (**B**, N = 15). **(C, D)** Analysis of the α-diversity by independent t test showed that the Chao 1 index [**C**, t(28) = −3.948, p = 0.000] and Sobs index [**D**, t(28) = −3.416, p = 0.002] was lower in tree shrews than humans. Error bars show the SEM. **p <0.01, ***p <0.001.

To preliminarily characterize the differences of gut microbiota composition and bio-diversity between healthy tree shrews and humans, we enrolled 15 healthy people from fmtBank and collected their fecal samples for 16S rRNA and did the same for 15 tree shrews. The results showed that humans had thirteen phyla and tree shrews had ten phyla. There were seven common phyla, including *Firmicutes*, *Proteobacteria*, *Actinobacteria*, *Bacteroidetes*, *Fusobacteria*, *Tenericutes* and *Verrucomicrobia*. Similarities and differences were observed between humans and tree shrews with probiotic implications. Humans had a higher relative abundance of *Bacteroidetes*, while tree shrews seemed to have a lower and higher relative abundance of *Bacteroidetes* and *Proteobacteria* respectively (**Figures 3A, B** **)**. The basic structure of microbiome in healthy humans is more anti-stress than healthy tree shrews. The α-diversity of tree shrew gut microbiota demonstrated that they have a poorer microbiota biodiversity compared to humans (**Figures 3C, D** **)**.

### WMT Reversed the Effects of Staying Up Late on Physiological Parameters Partially and the Changes on Brain Structure Effectively

**Figure 4:**
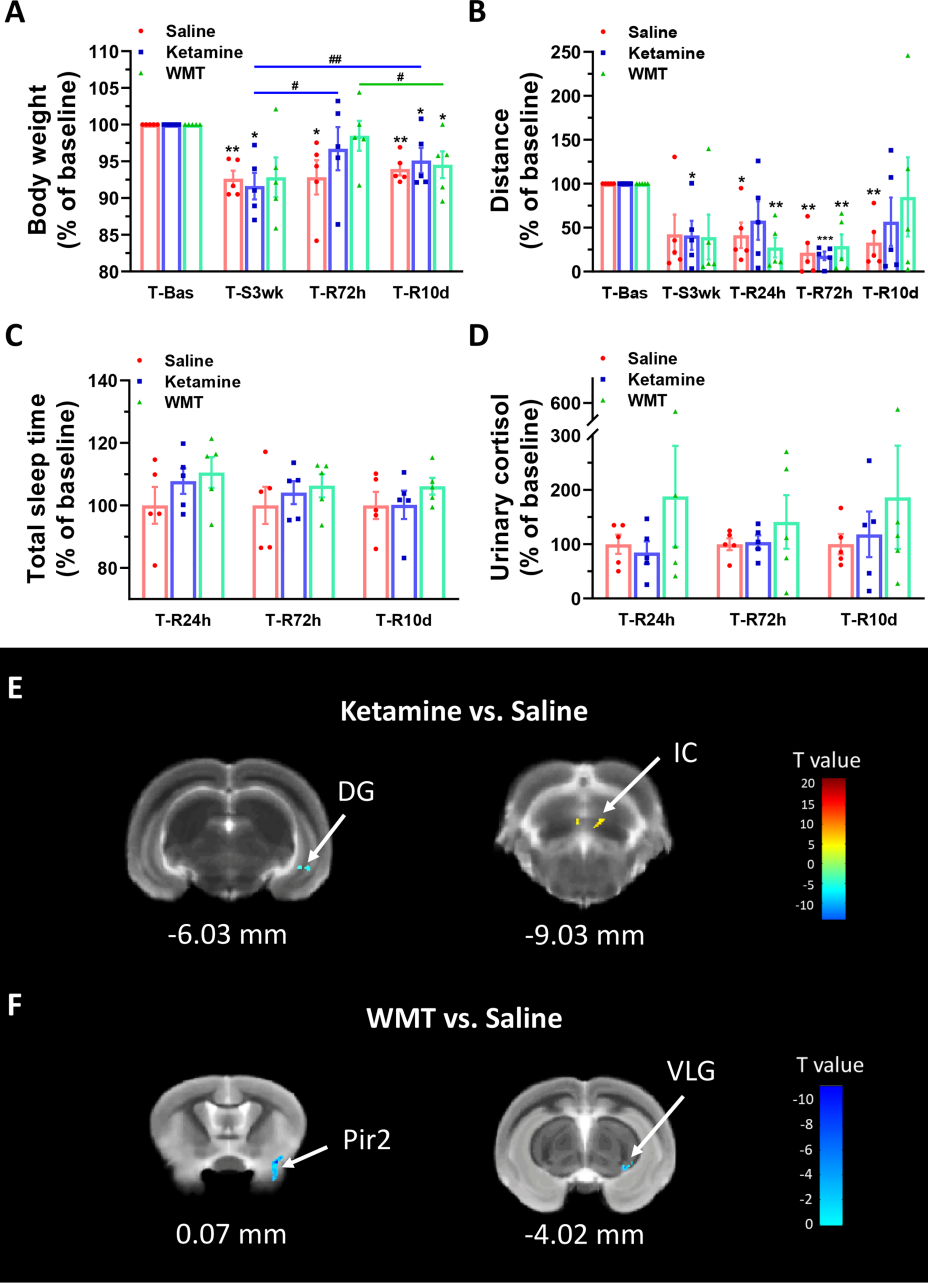
Physiological responses and changes in grey matter density of brain structure in tree shrews under different treatments during the recovery phase. **(A, B)** Body weight and distance travelled in the open field box under saline, ketamine and WMT. They were normalized with the values in the baseline (T-Bas) by using paired-samples t test. **(A)** In the saline group (the red column, N = 5), the body weight was decreased in T-S3wk [t(4) = 6.735, p = 0.003], T-R72h [t(4) = 3.075, p = 0.037] and T-R10d [t(4) = 7.093, p = 0.002]. In the ketamine group (the blue column, N = 5), the body weight was temporarily recovered at T-R72h to a baseline level and was improved compared to T-S3wk [t(4) = 3.509, p = 0.025], then dropped a bit yet was still higher at T-R10d [t(4) = 6.945, p = 0.002] compared to T-S3wk. In the WMT group (the green column, N = 5), tree shrews gained more weight in T-R72h than T-R10d [t(4) = 4.916, p = 0.008]. **(B)** In the saline group (the red column, N = 5), distance travelled in the open field box was decreased in T-R24h [t(4) = 4.013, p = 0.016], T-R72h [t(4) = 6.585, p = 0.003] and T-R10d [t(4) = 5.216, p = 0.006]. In the ketamine group (the blue column, N = 5), the distance was decreased in T-S3wk [t(4) = 3.532, p = 0.024] and T-R72h [t(4) = 17.117, p = 0.000]. In the WMT group (the green column, N = 5), the distance showed a decrease in T-R24h [t(4) = 6.457, p = 0.003] and T-R72h [t(4) = 5.293, p = 0.006]. **(C, D)** Total sleep time (C, N = 5/group) and morning urinary cortisol (D, N = 5/group) of tree shrews under saline, ketamine and WMT treatments. They were normalized by comparing with the saline group to avoid errors from humans and instruments. **(E)** The signal from DG (dentate gyrus of the hippocampus) decreased but that from IC (inferior colliculus) increased after ketamine administration (N = 3/group). **(F)** The signals from Pir2 (piriform cortex, layer 2) and VLG (ventral lateral geniculate nucleus) decreased after WMT (N = 3/group). Error bars show the SEM. *means compared with control in baseline phase, *p <0.05, **p <0.01, ***p <0.001. # means compared between the stress and recovery phase, #p <0.05, ##p <0.01.

We investigated whether WMT can remediate the alterations brought on by extended environmental lighting. Because healthy tree shrews are already prone to stress and have a higher baseline of *Proteobacteria*, the fecal microbiota from one human donor was administrated to light-stressed tree shrews. The enriched microbiota by washing process was prepared from one defecation of one healthy donor which was primarily screened for clinical medicine (Fecal Microbiota Transplantation-standardization Study, 2020; Zhang et al., 2020). To compare the therapeutic effect of WMT, ketamine, a rapid antidepressant (Berman et al., 2000), was used as the positive control and saline was used as the negative control. Body weight and distance travelled continuously decreased in the saline group (**Figures 4A, B** **)**. Considering that the change of body weight is a long-term process, we only measured body weight at the end of 3 weeks of extended light exposure and 72 h after treatment administration. The ketamine group had a fast recovery of body weight 72 h after administration [T-R72h compared with T-Bas: t(4) = 1.115, p = 0.327, **Figure 4A**] and the distance travelled improved, then dipped, then improved again 24 h, 72 h and 10 days after administration respectively [T-R24h compared with T-Bas: t(4) = 1.913, p = 0.128; T-R10d compared with T-Bas: t(4) = 1.572, p = 0.19, **Figure 4B**]. Moreover, the WMT group showed a similar recovery efficacy as ketamine, with a quick recovery of body weight 72 h after treatment [T-R72h compared with T-Bas: t(4) = 0.746, p = 0.497, **Figure 4A**]. The improvement of locomotion of tree shrews after WMT took longer and returned to baseline after 10 days [T-R10d compared with T-Bas: t(4) = 0.333, p = 0.756, **Figure 4B**]. The total sleep time and urine cortisol levels in both ketamine and WMT groups statistically did not differ from the saline group (**Figures 4C, D** **)**. However, the sleep onset time in WMT group was 1 h earlier than that in saline and ketamine groups (**Table S3**). Our analyses showed that ketamine and WMT had partial positive effects on the unfavorable influences of staying up late.

**Figure 5:**
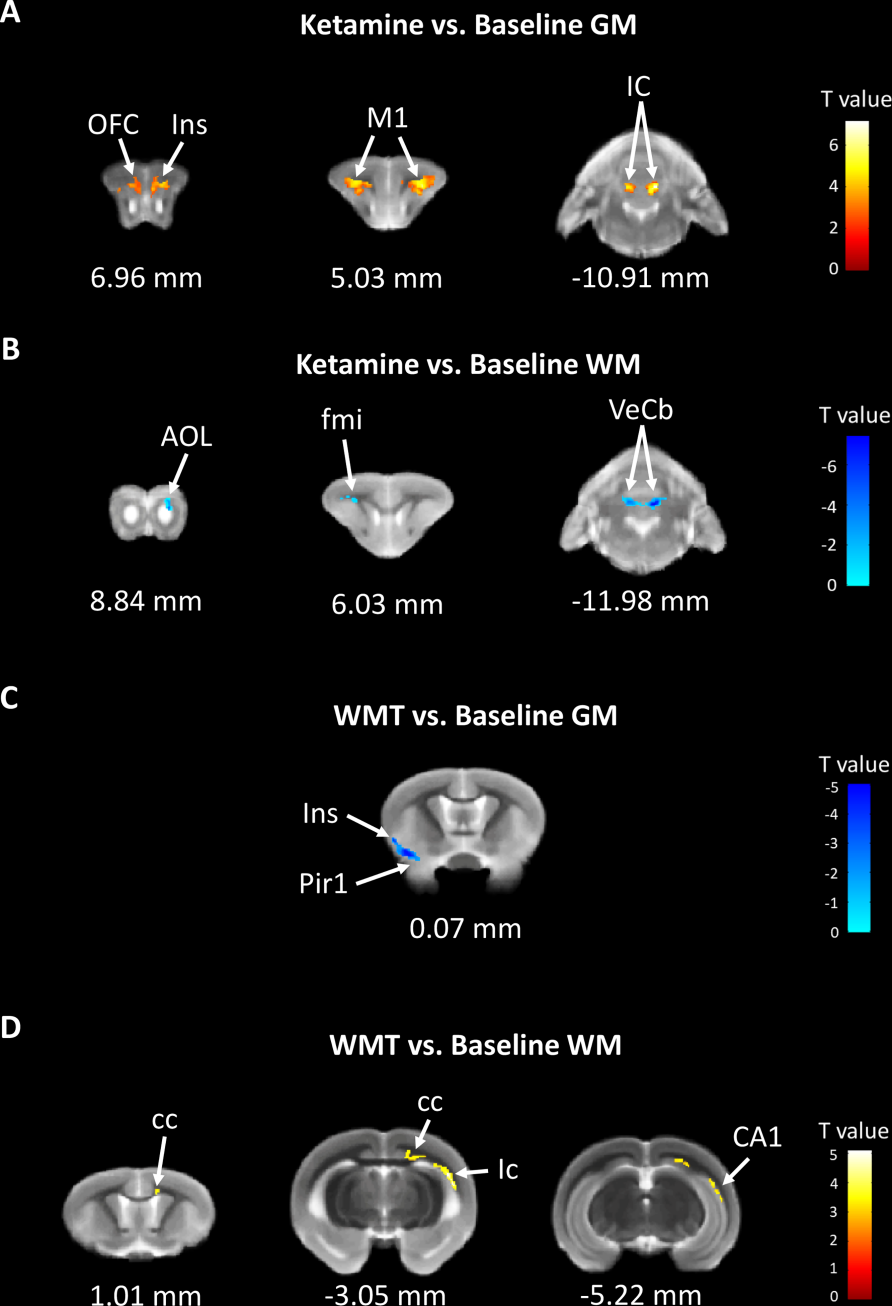
The changes in brain structure showed the recovery effect of WMT but potential addiction effect of ketamine. **(A)** The GM density signals from OFC (orbital frontal cortex), Ins (insular cortex), M1 (primary motor cortex) and IC (inferior colliculus) were increased, but **(B)** the WM density signals from AOL (anterior olfactory nucleus, lateral part), fmi (forceps minor of the corpus callosum) and VeCb (vestibulocerebellar nucleus) were decreased after ketamine administration. **(C)** The GM density signals from Ins and Pir1 (piriform cortex, layer 1) were decreased, but **(D)** the WM density signals from cc (corpus callosum), ic (internal capsule) and CA1 (field CA1 of the hippocampus) were increased after WMT. N = 3/group.

In order to characterize the therapeutic and recovery effects of ketamine and WMT, we performed two kinds of MRI comparisons. One pair is between the ketamine or WMT group and saline group, the other is between the ketamine or WMT group and baseline. Firstly, by comparing with the saline group, it was exhibited that the GM density of the ketamine group had a decrease in the hippocampus and an increase in the thalamus (**Figure 4E** and **Table 2A**). The signals of GM in the piriform cortex and lateral geniculate nucleus (LGN) decreased significantly in the WMT group (**Figure 4F** and **Table 2B**). Secondly, by comparing with the baseline, it was found that the GM and WM density changes by the light-induced stress in the frontal cortex, thalamus and corpus callosum still existed in the ketamine group, but disappeared in the WMT group (**Figure 5** and **Table 3**). New changes in the motor cortex (**Figure 5A** and **Table 3A**), anterior olfactory nucleus and cerebellum (**Figure 5B** and **Table 3A**) were observed in the ketamine group. Although the low dose of ketamine did not induce a phenotypic addictive response, the structural changes in the brain suggest that there might still be a slight risk of addiction. Note, that by comparing between the WMT group and baseline, we found that the trend of the whole brain density change was opposite to that after light-induced stress (**Figures 1F, G** and **Table 1**). The GM density in insular cortex and piriform cortex showed a significant decrease after WMT (**Figure 5C** and **Table 3B**). The WM density in corpus callosum, internal capsule and hippocampus were significantly increased after WMT (**Figure 5D** and **Table 3B**). The results indicated that WMT could contribute to the recovery-like effects for staying up late in tree shrews.

**Table 2:** The change of grey matter density in ketamine and WMT groups compared with saline group.

A							
Brain region	Cluster location	Coordinate			Cluster size (mm^3^)	T value	P
		x	y	z			
**Increase**							
Thalamus	**IC**	−1	7	−9	2.90	8.73	0.000^***^
**Decrease**							
Hippocampus	**DG**	−6	10	−6	3.18	14.00	0.000^***^
**B**							
Brain region	Cluster location	Coordinate			Cluster size (mm^3^)	T value	P
		x	y	z			
**Decrease**							
Piriform cortex	**Pir2**	−5	10	0	3.20	11.09	0.000^***^
LGN	**VLG**	−4	9	−4		8.65	0.000^***^

(A) Changed GM density of ketamine group compared with saline group. (B) Changed GM density of WMT group compared with saline group. ***p <0.001.

**Table 3:** Brain regions changes in tree shrews with the treatment of ketamine and WMT by compared with baseline phase.

A							
Brain region	Cluster location	Coordinate			Cluster size (mm^3^)	T value	P
		x	y	z			
**Increase**							
Insular cortex	**Ins**	3	5	7	8.16	3.92	0.001^**^
Motor cortex	**M1**	−4	4	5	9.44	3.55	0.002^**^
Thalamus	**IC**	−1	7	−11	3.96	7.14	0.000^***^
**Decrease**							
Anterior olfactory nucleus	**AOL**	−2	6	9	2.46	7.07	0.000^***^
Corpus callosum	**fmi**	3	4	6	2.93	5.31	0.000^***^
Cerebellum	**VeCb**	−1	8	−12	4.49	7.44	0.000^***^
**B**							
Brain region	Cluster location	Coordinate			Cluster size (mm^3^)	T value	P
		x	y	z			
**Decrease**							
Insular cortex	**Ins**	7	8	0	3.42	3.82	0.001^**^
Piriform cortex	**Pir1**	6	9	−0		36.9	0.002^**^
**Increase**							
Corpus callosum	**cc**	−2	3	1	4.97	4.07	0.001^**^
Internal capsule	**ic**	−7	6	−3	3.30	5.08	0.000^***^
Hippocampus	**CA1**	−7	6	−5		3.67	0.002^**^

(A) Changed GM and WM density of ketamine group compared with baseline. (B) Changed GM and WM density of FMT WMT group compared with baseline. **p < 0.01, ***p < 0.001.

## Discussion

The present study successfully established a staying up late model in tree shrew by prolonging the light at night for 4 h. The behaviors, microbiota composition and brain signals were changed by the light-induced stress. Part of these alterations could be rescued by WMT. The present finding highlighted that tree shrews are light-stress sensitive animal model with close microbiota composition between the healthy tree shrews and humans.

### Staying Up Late Model

With the advent of the incandescent bulb, unnaturally timed artificial light from the environment can and has disrupted our biological rhythms (Bedrosian and Nelson, 2013). To study this, we developed a staying up late model. From the point of view of sleep deprivation, it expands on the various ways of sleep depriving to accurately mimic the situation progressively more persons find themselves in (Colavito et al., 2013). The animal was not forced per se to stay awake. Light was just kept on longer and by consequence total sleep time decreased and was thus sleep deprived (Bandyopadhyay and Sigua, 2019). The extended light exposure at night resulted in a body weight loss, reduced locomotor activities, a loss of total sleep time and an elevated cortisol level, and differences in microbiota composition illustrating the drastic effects of an otherwise simple environmental change. This model can assist further explorations on how our modern lifestyle affects our health. It could aid studies of sleep, rhythm disturbances and stress as both dimensions reside in this model. We speculate that to a (limited) extent it might even be informative for depression research as aforementioned factors intersect with depression. Some similar symptoms of the model have been described in a chronic psychosocial stress model of tree shrews (Fuchs et al., 2001; Wang et al., 2013a; Fang et al., 2016). There, male tree shrews are used and show depressive-like symptoms due to social defeat (Meyer et al., 2001). It requires tree shrews to fight with each other, and is at times variable and unpredictable. The staying up late model is based on controllable light exposure and straightforward.

### Light-Induced Stress and Microbiota

The Earth’s rotation brings about a cyclical light-dark phase change, on which almost all living organisms have adapted their physiology in sophisticated fashions. Fiddling with it and extended light exposure at night can then disrupts circadian rhythms leading to, in humans, the prevalence of psychiatric and behavioral disorders (Lambert et al., 2015). Along the gut-brain axis a recent investigation revealed that 24 h of continuous light- or dark-induced stress for 12 weeks influenced the memory and the composition of gut microbiota in mice (Kim et al., 2019). The observations of Cui et al. showed that prolonging the time of exposure to light could shape the gut microbiota composition in mice (Cui et al., 2016). Meanwhile, the gut microbiota, in turn, regulated the circadian rhythm of host metabolism (Kuang et al., 2019). Consistent with this, our observation showed that significant changes in the gut microbiota composition were found after only 4 h extended light exposure in tree shrews.

*Firmicutes* and *Bacteroidetes* present the main groups in gut microbiota (Kelly et al., 2016). Studies revealed that there was a decrease in *Firmicutes* but an increase in *Proteobacteria* in irritable bowel syndrome (IBS) and major depressed patients. The ratio of the *Firmicutes*-to-*Bacteroidetes* was also increased in IBS patients compared with healthy controls (Krogius-Kurikka et al., 2009; Inserra et al., 2018). Similarly, tree shrews under light-induced stress for one week showed a decrease of *Firmicutes*, an increase of *Proteobacteria*, and a higher ratio of *Firmicutes*-to-*Bacteroidetes*. Recent evidences showed that microbiota played a key role in circulating metabolites by altering the production of short-chain fatty acids (Komaroff, 2017; Vojinovic et al., 2019). A study comparing the microbiota of obese children with that of lean children provided evidence that the *Firmicutes*-to-*Bacteroidetes* ratio of obese children was much higher (Riva et al., 2017). Therefore, the reduction of the ratio of the *Firmicutes*-to-*Bacteroidetes* might be related to the body weight loss at the end of light-induced stress phase in tree shrews.

### Gut Microbiota Changed the Structure of the Brain

Lines of evidence in both clinical and animal research suggest that the gut–brain axis can be a new therapeutic angle for central nervous system disorders (Long-Smith et al., 2020). Though few pilot studies observed the role of WMT on typical brain diseases (Xu et al., 2021), such as epilepsy (He et al., 2017), little is known on how WMT could alter brain structure. By comparing the GM density before and after the light-induced stress in our model, the MRI results revealed an increase in environmental light affected the frontal cortex and sensory system, particularly the visual system. Interestingly, these changes disappeared after WMT. This was accompanied by a robust decrease in the GM density from the insular and piriform cortex. The insular cortex is involved in consciousness in humans, and plays a role in emotion regulation. Evidence from rhesus monkeys revealed that the insular cortex was associated with the amygdala (Mufson et al., 1981). There is evidence that the posterior insula connects with the somatosensory cortex and involves in audio–visual integration tasks (Bushara et al., 2001). The piriform cortex, mainly contributing to ensemble coding of odor, was located between the insular cortex and the anteriorly and laterally of the amygdala in humans (Howard et al., 2009). Surprisingly, the anatomy of this location in tree shrews was strikingly similar to the humans, evincing of the close relationship of tree shrews to primates in regard of brain structure.

The WM density of corpus callosum, internal capsule and hippocampus increased significantly between the WMT treatment and the baseline, which is consilient with previous engrossing explorations. The study from Cryan’s lab indicated that the microbiota was necessary for appropriate cortical myelination at an ultrastructural level (Hoban et al., 2016). Gut dysbiosis in neonatal C57BL/6 mice can potentially alter myelination and thus impair cognition (Keogh et al., 2021). This provides direct evidence that gut microbiota plays an essential role in basic neurogenerative processes.

Additionally, this study compared the therapeutic effect of different treatments on the brain structure, and proved that ketamine treatment mainly affects the hippocampus (Price and Duman, 2020). The present findings showed that WMT mainly affected the piriform cortex and LGN. Therefore, ketamine acted different way from WMT in brain, at least in light-induced stress model.

In conclusion, we successfully established a staying up late model in tree shrews to explore the effects of extended light exposure on physiology, behavior and brain structure. Our findings indicated that this model could be informative for researching stress-sensitive diseases and that the strategy of targeting the gut-brain axis could perhaps one day bare therapeutic fruit.

## Data Availability Statement

The original contributions presented in the study are included in the article/**Supplementary Material**. Further inquiries can be directed to the corresponding authors.

## Ethics Statement

The studies involving human participants were reviewed and approved by the Second Affiliated Hospital of Nanjing Medical University Institutional Review Board. The patients/participants provided their written informed consent to participate in this study. The animal study was reviewed and approved by the Experimental Animal Ethics Committee at the Nanjing Medical University. Written informed consent was obtained from the individual(s) for the publication of any potentially identifiable images or data included in this article.

## Author Contributions

JW, JG and FZ designed the research. JW and ML participated in behavior and MRI experiments. PL and QL performed microbiota study and chemical tests. MZ prepared for the special open field box for tree shrews. JD and JX recorded the sleep video. JW, QL and QH analyzed data. JW, QL, FZ and JG drafted the manuscript. All authors contributed to the article and approved the submitted version.

## Funding

This work was financially supported by grants from the National Natural Science Foundation of China (81801314, 81973308, and 81600417), the Natural Science Foundation of the Jiangsu Higher Education Institutions of China (18KJB180016), the Key R&D Program of Jiangsu Province (2017CX010) and the Jiangsu Undergraduate Innovation and Entrepreneurship Program (201910312051Y).

## Conflict of Interest

FZ invented the concept of GenFMTer and devices related to it.

The remaining authors declare that the research was conducted in the absence of any commercial or financial relationships that could be construed as a potential conflict of interest.
