# Supplementary material for: Washed Microbiota Transplantation Accelerates the Recovery of Abnormal Changes by Light-Induced Stress in Tree Shrews
Source: Front Cell Infect Microbiol. 2021 Jun 23;11:685019. doi: 10.3389/fcimb.2021.685019 (PMC8262326; doi:10.3389/fcimb.2021.685019)
Supplement: Supplementary file 1 [file DataSheet_1.pdf]

Table S1. The time of sleep onset and waking up after extended light exposure for 1 week and 3 weeks.

Items	T-Bas	T-S1wk	T- S3wk
Sleep onset time	20:06±0:02	21:50±0:18	21:43±0:14
Wake up time	7:44±0:03	7:56±0:01	7:54±0:02
Total sleep time	11:18±0:07	9:45±0:16	9:45±0:13

Table S2. Correlation analysis between changes of gut microbiota and alterations of physiological responses in tree shrews.

Level	Microbiota	95%CI	r	P
		**Weight**		
Phylum	***Tenericutes***	0.078 to 0.846	0.579	0.026*
	Lautropia	-0.877 to -0.194	-0.652	0.010*
	Leptotrichia	-0.831 to -0.026	-0.543	0.039*
	Haemophilus	-0.825 to -0.006	-0.529	0.045*
Genus	***Bergeyella***	-0.842 to -0.061	-0.568	0.030*
	Aggregatibacter	-0.885 to -0.228	-0.672	0.007**
	Lysinibacillus	0.007 to 0.825	0.529	0.043*
	Anaerovibrio	0.004 to 0.824	0.527	0.044*
		**Distance**		
	Kurthia	0.267 to 0.894	0.694	0.002**
	Atopostipes	0.120 to 0.858	0.6061	0.018*
Genus	***Moraxella***	0.088 to 0.849	0.586	0.019*
	Finegoldia	0.097 to 0.852	0.592	0.0095**
	Methylobacterium	-0.826 to -0.009	-0.531	0.044*
		**Urinary cortisol**		
Phylum	***Deferribacteres***	0.221 to 0.883	0.668	0.006**
	Senegalimassilia	0.134 to 0.862	0.615	0.015*
	Shuttleworthia	0.006 to 0.825	0.529	0.045*
Genus	***Ureaplasma***	0.004 to 0.824	0.527	0.045*
	Mucispirillum	0.221 to 0.883	0.668	0.006**
	Anaerofustis	-0.015 to 0.818	0.514	0.0498*
	Olsenella	-0.850 to -0.089	-0.586	0.019*

Continued from last page

Level	Microbiota	95%CI	r	P
	**Sleep**			
	Phascolarctobacterium	0.170 to 0.870	0.636	0.012*
	Parabacteroides	0.123 to 0.859	0.608	0.018*
	Trichococcus	0.007 to 0.824	0.529	0.045*
	Alistipes	0.113 to 0.856	0.602	0.020*
	Holdemania	0.163 to 0.869	0.633	0.0095**
Genus	***Anaerofustis***	0.102 to 0.853	0.595	0.019*
	Streptococcus	-0.872 to -0.173	-0.639	0.012*
	Lachnoclostridium	-0.861 to -0.132	-0.614	0.017*
	Johnsonella	-0.883 to -0.222	-0.669	0.008**
	Tannerella	-0.876 to -0.192	-0.651	0.009**
	Propionivibrio	-0.846 to -0.076	-0.577	0.025*

The alterations of relative abundance in phylum *Tenericutes* and genera *Lysinibacillus* and *Anaerovibrio* were positively associated with the changes in body weight before and three weeks after stress. Further analysis revealed a negative correlation between the changes of relative abundance and body weight, including genera *Lautropia, Leptotrichia, Haemophilus, Bergeyella* and *Aggregatibacter*.

There was a significantly positive correlation between the changes of gut microbiota relative abundance and activities before and three weeks after stress, including the genera *Kurthia, Atopostipes, Moraxella* and *Finegoldia*. However, the changes relative abundance of *Methylobacterium* were negatively related to the changes in activities.

A positive correlation was observed between the alterations of relative abundance in phylum *Deferribacteres* and genera *Senegalimassilia, Shuttleworthia, Ureaplasma, Mucispirillum* and *Anaerofustis* and the changes of urinary cortisol before and after stress. A significantly negative correlation was found between the alterations of relative abundance in genera *Olsenalla* and the changes of urinary cortisol.

The most important is that an apparent correlation was discovered between the changes of gut microbiota relative abundance and the total sleep time before and three weeks after stress. The positive correlation was observed in genera *Phascolarctobacterium, Parabacteroides, Trichococcus, Alistipes, Holdemania* and *Anaerofustis* and the negative correlation was observed in genera *Streptococcus, Lachnoclostridium, Johnsonella, Tannerella* and *Propionivibrio*. * $p < 0.05$, ** $p < 0.01$.

Table S3. The time of sleep onset and waking up under extended light exposure after different treatments.

Items	Groups	T-R24h	T-R72h	T-R10d
	Saline	22:07±0:37	22:37±0:35	22:25±0:28
**Sleep onset time**	Ketamine	21:31±0:27	22:03±0:23	22:32±0:26
	WMT	21:36±0:24	22:06±0:18	21:21±0:09
	Saline	7:50±0:03	7:58±0:00	7:54±0:07
**Wake up time**	Ketamine	8:08±0:06	7:57±0:01	8:02±0:02
	WMT	8:06±0:07	7:59±0:06	7:56 ±0:04
	Saline	9:26±0:33	9:09±0:32	9:08±0:23
**Total sleep time**	Ketamine	10:10±0:23	9:32±0:20	9:09±0:24
	WMT	10:25±0:27	9:44±0:20	9:41±0:14
